# HIV Denial in the Internet Era

**DOI:** 10.1371/journal.pmed.0040256

**Published:** 2007-08-21

**Authors:** Tara C Smith, Steven P Novella

## Abstract

The Internet has served as a fertile and un-refereed medium to spread HIV denialist beliefs, argue the authors.

It may seem remarkable that, 23 years after the identification of the human immunodeficiency virus (HIV), there is still denial that the virus is the cause of acquired immune deficiency syndrome (AIDS). This denial was highlighted on an international level in 2000, when South African president Thabo Mbeki convened a group of panelists to discuss the cause of AIDS, acknowledging that he remained unconvinced that HIV was the cause [1]. His ideas were derived at least partly from material he found on the Internet [2]. Though Mbeki agreed later that year to step back from the debate [3], he subsequently suggested a re-analysis of health spending with a decreased emphasis on HIV/AIDS [4].

HIV denial has taken root in the general population and has shown its potential to frustrate public education efforts and adversely affect public funding for AIDS research and prevention programs. For example, the AIDS Coalition to Unleash Power (ACT UP) was for many years on the front lines of AIDS education and activism. But now a San Francisco chapter of the group has joined the denialist movement, stating on its Web site that “HIV does not cause AIDS… HIV antibody tests are flawed and dangerous…AIDS drugs are poison” (http://www.actupsf.com/aids/index.htm). In 2000 the chapter wrote letters to every member of Congress asking them to stop funding research into HIV [5]. ACT UP San Francisco's position has been condemned by other ACT UP chapters, such as ACT UP Philadelphia and ACT UP East Bay (http://www.actupny.org/indexfolder/actupgg.html). Rock stars have weighed in on the topic. Members of the group “The Foo Fighters” provided music for a soundtrack of the recent documentary, “The Other Side of AIDS” (http://www.theothersideofaids.com/), which questions whether HIV is the cause of AIDS. The band has spread its message that HIV does not cause AIDS at concerts [6], and it lists the HIV denial group “Alive and Well” as a worthy cause on its Web site (http://www.foofighters.com/community_cause.html).

As these challenges to mainstream theories have largely occurred outside of the scientific literature, many physicians and researchers have had the luxury of ignoring them as fringe beliefs and therefore inconsequential. Indeed, the Internet has served as a fertile and un-refereed medium to spread these denialist beliefs. The Group for the Scientific Reappraisal of the HIV/AIDS Hypothesis (“Reappraising AIDS”) noted, “Thanks to the ascendance of the internet, we are now able to reinvigorate our informational campaign” [7]. The Internet is an effective tool for targeting young people, and for spreading misinformation within a group at high risk for HIV infection.

Two excellent online fact sheets have been prepared to counter many of the most commonly used arguments to deny HIV causation of AIDS [8,9]; as such, we will not discuss these in this article. Instead, we will review the current intellectual strategies used by the HIV denial movement. Although other forms of science denial will not be specifically discussed, the characteristics described below apply to many other forms of popular denial, including denial of evolution, mental illness, and the Holocaust.

## Three Prominent Deniers and Denial Groups

One of the prominent HIV denial groups currently is Christine Maggiore's “Alive and Well” (formerly “HEAL,” Health Education AIDS Liaison) (http://www.aliveandwell.org/). Maggiore's life story is at the center of this group. Diagnosed with HIV in 1992, Maggiore claims she has since been symptom-free for the past 14 years without the use of antiretroviral drugs, including protease inhibitors [10]. She has risen to prominence, and been embroiled in controversy, in recent years after giving birth to and openly breast-feeding her two children, Charles and Eliza Jane. She had neither child tested for HIV, and did not take antiretroviral medication during her pregnancy or subsequent breast-feeding [11]. Eliza Jane died in September 2005 of HIV-related pneumonia [12], though Maggiore remains unconvinced that HIV had any role in her daughter's death [13], and continues to preach her message to other HIV-positive mothers.

Peter Duesberg initiated the HIV denial movement with a 1987 article suggesting that HIV does not cause AIDS [14]. While he is no longer on the front lines of this movement, the arguments put forth by others trace back to his publications.

Celia Farber is a journalist who has spent much of her career covering HIV. Farber is the author of a recent *Harper's* article repeating Duesberg's claims that HIV does not cause AIDS [15], and has recently authored a book on “the shadowy story of AIDS science” [16].

There are serious inconsistencies within the broad HIV denial movement, and the individuals mentioned above are only the tip of the iceberg. HIV denial groups diverge even on the most basic tenet: does HIV exist at all? Nevertheless, disagreements within the movement are overlooked for the sake of presenting a unified front.

## Conspiracy Theories and Selective Distrust of Scientific Authority

That HIV is the primary cause of AIDS is the strongly held consensus opinion of the scientific community, based upon over two decades of robust research. Deniers must therefore reject this consensus, either by denigrating the notion of scientific authority in general, or by arguing that the mainstream HIV community is intellectually compromised. It is therefore not surprising that much of the newer denial literature reflects a basic distrust of authority and of the institutions of science and medicine. In her book, Christine Maggiore thanks her father Robert, “who taught me to question authority and stand up for what's right” [10]. Similarly, mathematical modeler Dr. Rebecca Culshaw, another HIV denier, states: “As someone who has been raised by parents who taught me from a young age never to believe anything just because ‘everyone else accepts it to be true,’ I can no longer just sit by and do nothing, thereby contributing to this craziness” [17].

Distrusting mainstream medical practitioners, many HIV deniers turn to “alternative” medicine in search of treatment. One such practitioner, Dr. Mohammed Al-Bayati, suggests that “toxins” and drug use, rather than HIV, cause AIDS [18]. Dr Al-Bayati personally profits from his HIV denialism: for $100 per hour, Al-Bayati will consult “on health issues related to AIDS, adverse reactions to vaccines and medications, exposure to chemicals in the home, environment or workplace” (http://www.toxi-health.com/). Similarly, German vitamin supplier and HIV denier Matthias Rath not only pushed his vitamins as a treatment for AIDS [19], but his spokesman refused to be interviewed by *Nature Medicine* about the case because he claimed the journal is “funded to the hilt with drug money” [20].

Deniers argue that because scientists receive grant money, fame, and prestige as a result of their research, it is in their best interest to maintain the status quo [15]. This type of thinking is convenient for deniers as it allows them to choose which authorities to believe and which ones to dismiss as part of a grand conspiracy. In addition to being selective, their logic is also internally inconsistent. For example, they dismiss studies that support the HIV hypothesis as being biased by “drug money,” while they accept uncritically the testimony of HIV deniers who have a heavy financial stake in their alternative treatment modalities.

## Portraying Science as Faith and Consensus as Dogma

Since the ideas proposed by deniers do not meet rigorous scientific standards, they cannot hope to compete against the mainstream theories. They cannot raise the level of their beliefs up to the standards of mainstream science; therefore they attempt to lower the status of the denied science down to the level of religious faith, characterizing scientific consensus as scientific dogma [21]. As one HIV denier quoted in Maggiore's book [10] remarked,

*“There is classical science, the way it's supposed to work, and then there's religion. I regained my sanity when I realized that AIDS science was a religious discourse. The one thing I will go to my grave not understanding is why everyone was so quick to accept everything the government said as truth. Especially the central myth: the cause of AIDS is known.”*

Others suggest that the entire spectrum of modern medicine is a religion [22].

Deniers also paint themselves as skeptics working to break down a misguided and deeply rooted belief. They argue that when mainstream scientists speak out against the scientific “orthodoxy,” they are persecuted and dismissed. For example, HIV deniers make much of the demise of Peter Duesberg's career, claiming that when he began speaking out against HIV as the cause of AIDS, he was “ignored and discredited” because of his dissidence [23]. South African President Mbeki went even further, stating: “In an earlier period in human history, these [dissidents] would be heretics that would be burnt at the stake!” [1].

HIV deniers accuse scientists of quashing dissent regarding the cause of AIDS, and not allowing so-called “alternative” theories to be heard. However, this claim could be applied to any well-established scientific theory that is being challenged by politically motivated pseudoscientific notions—for example, creationist challenges to evolution. Further, as HIV denial can plausibly reduce compliance with safe sex practices and anti-HIV drugs, potentially costing lives, this motivates the scientific and health care communities to exclude HIV denial from any public forum. (As one editorial has bluntly phrased it, HIV denial is “deadly quackery”) [24]. Because HIV denial is not scientifically legitimate, such exclusion is justified, but it further fuels the deniers' claims of oppression.

## Expert Opinion and the Promise of Forthcoming Scientific Acceptance

Although the HIV deniers condemn scientific authority and consensus, they have nevertheless worked to assemble their own lists of scientists and other professionals who support their ideas. As a result, the deniers claim that they are just on the cusp of broader acceptance in the scientific community and that they remain an underdog due to the “established orthodoxy” represented by scientists who believe that HIV causes AIDS.

In an effort to support its claim that an increasing number of scientists do not believe that HIV causes AIDS, Reappraising AIDS has published a list of signatories agreeing to the following statement:

“It is widely believed by the general public that a retrovirus called HIV causes the group [of] diseases called AIDS. Many biochemical scientists now question this hypothesis. We propose that a thorough reappraisal of the existing evidence for and against this hypothesis be conducted by a suitable independent group. We further propose that critical epidemiological studies be devised and undertaken” [25].

These signatories do not, however, suggest who the “suitable independent” group should be, since, presumably, many scientists have already been “indoctrinated” into believing that HIV causes AIDS. (Indeed, many of the signatories to this statement lack any qualifications in virology, epidemiology, or even basic biology.) They also ignore thousands of epidemiological studies that have already been published in the scientific literature. And the signatories fail to provide a convincing case that there is widespread acceptance in the scientific community for their marginal position.

Nevertheless, Farber wrote in a 1992 article that “more and more scientists are beginning to question the hypothesis that HIV single-handedly creates the chaos in the immune system that leads to AIDS” [26]. Similarly, a March 2006 article appearing on the AIDS denial Web site “New AIDS Review” claims that, in reference to the theory that HIV causes AIDS: “…the fabric of this theoretical mantle is threadbare to the point of disintegration” [27]. Mainstream scientists, of course, do not believe in the imminent demise of the HIV theory; instead they continue to produce novel research on preventing and treating HIV and publish thousands of papers every year on the topic.

Further, deniers exploit the sense of fair play present in most scientists, and also in the general public, especially in open and democratic societies. Calling for a fair discussion of dissenting views, independent analysis of evidence, and openness to alternatives is likely to garner support, regardless of the context. But it is misleading for the HIV denial movement to suggest that there is any real doubt about the cause of AIDS.

## Pushing Back the Goalpost

Of all the characteristics of deniers, repeatedly nudging back the goalpost—or the threshold of evidence required for acceptance of a theory—is often the most telling. The strategy behind goalpost-moving is simple: always demand more evidence than can currently be provided. If the evidence is then provided at a later date, simply change the demand to require even more evidence, or refuse to accept the kind of evidence that is being offered.

In the 1980s, HIV deniers argued that drug therapy for AIDS was ineffective, did not significantly prolong survival, and in fact was toxic and damaged the immune system [28]. However, after the introduction of a cocktail of newer and more effective agents in the 1990s, survival rates did impressively increase [29]. HIV deniers no longer accept this criterion as evidence for drug effectiveness, and therefore the HIV theory of AIDS. Even stacks of papers and books published on the subject are not enough. Christine Maggiore writes in her book, “Since 1984, more than 100,000 papers have been published on HIV. None of these papers, singly or collectively, has been able to reasonably demonstrate or effectively prove that HIV can cause AIDS” [10].

HIV deniers also arbitrarily reject categories of evidence, even though they are generally accepted across scientific disciplines. For example, they deny inferential evidence that HIV causes AIDS, including data examining the closely related simian immunodeficiency virus (SIV) in genomic and animal studies [30]. Likewise they reject correlation as insufficient to establish causation [28]. However, multiple independent correlations pointing to the same causation—in this case that HIV causes AIDS—is a legitimate and generally accepted form of epidemiological evidence used to establish causation. The same type of evidence, for example, has been used to establish that smoking causes certain types of lung cancer.

## What Are Their Alternatives?

After so much criticism levied upon the prevailing theories by deniers, one might think they would have something to offer to replace HIV as the cause of AIDS. However, the alternatives they offer are much more speculative than the mainstream theories they decry as lacking evidence. Further, their arguments amount to little more than another logical fallacy, the false dichotomy: they assume that overturning the prevailing theory will prove their theory correct, by default.

Interestingly, alternative hypotheses for AIDS causation depend on where the patient lives. In Africa, HIV deniers attribute AIDS to a combination of malnutrition and poor sanitation, i.e., they believe that AIDS is simply a relabeling of old diseases. In America and other wealthy countries, they claim AIDS is caused by drug use and promiscuity. Duesberg has long been an advocate of the idea that the use of “poppers,” or amyl nitrate, is a cause of AIDS in the gay community [31]. With the identification of AIDS in individuals who have never used poppers, this hypothesis has been widened by HIV deniers to implicate a number of recreational drugs (cocaine, crack, heroin, methamphetamines) as well as prescription drugs such as antibiotics and steroids in the etiology of AIDS. HIV deniers have criticized the idea that immunosuppression due to infection with HIV could result in all of the different infections that characterize AIDS, and yet they support the idea that poppers or other drugs—including many that have not been shown to cause severe immune deficiencies—could cause AIDS. In the past decade, the very drugs used to treat HIV/AIDS have come under fire by HIV deniers, who have suggested that the medicines themselves are a cause of AIDS (http://www.aliveandwell.org/) .

## Conclusion

Because these denialist assertions are made in books and on the Internet rather than in the scientific literature, many scientists are either unaware of the existence of organized denial groups, or believe they can safely ignore them as the discredited fringe. And indeed, most of the HIV deniers' arguments were answered long ago by scientists. However, many members of the general public do not have the scientific background to critique the assertions put forth by these groups, and not only accept them but continue to propagate them. A recent editorial in *Nature Medicine* [32] stresses the need to counteract AIDS misinformation spread by the deniers.

While the descriptions of HIV denialism above refer to relatively organized campaigns, there are other less orchestrated examples of such denialism. A recent study, for example, showed that a large percentage of African Americans are suspicious of mainstream AIDS theories due to a general distrust of government authorities [33]. Arguments by denial groups may have played a role in the formation of their opinion. Indeed, the effect of denial groups on public perception of HIV infection is an area ripe for careful research, as this denial can have lethal consequences. In the recent study, stronger conspiracy beliefs were significantly associated with more negative attitudes towards using condoms and with inconsistent condom use, independent of selected sociodemographic characteristics, partner variables, sexually transmitted disease history, perceived risk, and psychosocial factors [33].

How much of this lingering denial is the fault of scientists and the media for originally proclaiming AIDS a universal “death sentence”? Even though this idea may no longer appear in the scientific literature, it remains a public perception of the disease. It is difficult to strike the correct balance between providing information conveying on one hand the severity of the disease, and on the other optimism about treatment and advances in understanding HIV pathogenesis (including research about individuals who may indeed be somewhat resistant to the virus). Oversimplifying AIDS science to the public lends itself to exploitation by AIDS deniers who remain “alive and well” years after diagnosis with HIV. Yet these concerns must be balanced with the desire to convey the proper gravity of the situation and motivate those who are known to be HIV positive to seek treatment: a difficult line to walk.

This balancing act, in fact, deserves increasing attention from medical scientists in the age of the Internet and a broadening gap between the practice of science and public understanding of science. Successful public health education requires the presentation of a clear and simple message supported by a solid consensus of the medical community. Yet the reality behind the scenes is often quite different. Every medical field has its legitimate controversies and complexities, and the process of science is often messy. Denial groups exploit the gap between public education and scientific reality.

Further, countering the misinformation of HIV deniers needs to be conducted in the broader societal context of countering anti-science and pseudoscience. The strategies of HIV deniers, like many other denialist movements, seek to undermine the very philosophy of science itself, to distort public understanding of the scientific process, and to sow distrust of scientific institutions. Unscientific alternative medical modalities have made significant inroads into the institutions of health care through political means, despite a continued lack of scientific legitimacy: vaccines are characterized as dangerous instead of life-saving; psychiatry is mocked by celebrities and others in the public eye. Meanwhile, many leaders in science and business are concerned that the United States is losing its edge as a scientific powerhouse.

There remains a deep problem of overall scientific illiteracy in this country and others, creating fertile soil for those who wish to spread scientific misinformation [34]. The scientific community must collectively defend and promote the role of science in society, and combat the growing problem of scientific illiteracy. We must all strive to do our part to make science accessible to the general public, and to explain the process by which scientific evidence is gathered, analyzed, and eventually accepted, and academic institutions should provide greater incentive for their researchers to expend the time and effort to do so. A solid understanding of the scientific method may not eliminate science denial, but it may act as a buffer against the further spread of such denialist beliefs.

**Figure:**
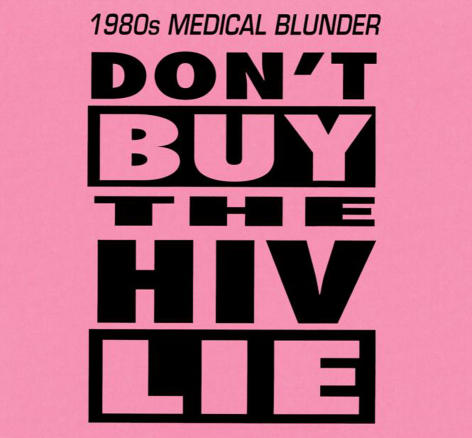
Example of a typical slogan from an HIV denialist group
